# Phylogenetic Conservation of Soil Microbial Responses to Elevated Tropospheric Ozone and Nitrogen Fertilization

**DOI:** 10.1128/msystems.00721-22

**Published:** 2023-01-10

**Authors:** Zhengsheng Yu, Qun Gao, Xue Guo, Jinlong Peng, Qi Qi, Xunwen Chen, Mengying Gao, Cehui Mo, Zhaozhong Feng, Ming Hung Wong, Yunfeng Yang, Hui Li

**Affiliations:** a Guangdong Provincial Research Centre for Environment Pollution Control and Remediation Materials, Department of Ecology, College of Life Science and Technology, Jinan University, Guangzhou, China; b State Key Joint Laboratory of Environment Simulation and Pollution Control, School of Environment, Tsinghua University, Beijing, China; c Key Laboratory of Ecosystem Network Observation and Modeling, Institute of Geographic Sciences and Natural Resources Research, Chinese Academy of Sciences, Beijing, China; d School of Applied Meteorology, Nanjing University of Information Science and Technology, Nanjing, China; University of Waterloo

**Keywords:** global tropospheric ozone, nitrogen fertilization, maize, microbial response, phylogenetic conservation, biomass reduction, crop, ozone

## Abstract

Plant primary productivity and crop yields have been reduced due to the doubled level of global tropospheric ozone. Little is known about how elevated ozone affects soil microbial communities in the cropland ecosystem and whether such effects are sensitive to the nitrogen (*N*) supply. Here, we examined the responses of bacterial and fungal communities in maize soils to elevated ozone (+60 ppb ozone) across different levels of *N* fertilization (+60, +120, and +240 kg *N* ha^−1^yr^−1^). The fungal alpha diversity was decreased (*P < *0.05), whereas the bacterial alpha diversity displayed no significant change under elevated ozone. Significant (*P < *0.05) effects of *N* fertilization and elevated ozone on both the bacterial and fungal communities were observed. However, no interactive effects between *N* fertilization and elevated ozone were observed for bacterial and fungal communities (*P > *0.1). The bacterial responses to *N* fertilization as well as the bacterial and fungal responses to elevated ozone were all phylogenetically conserved, showing universal homogeneous selection (homogeneous environmental conditions leading to more similar community structures). In detail, bacterial Alphaproteobacteria, Actinobacteria, and Chloroflexi, as well as fungal Ascomycota, were increased by elevated ozone, whereas bacterial Gammaproteobacteria, Bacteroidetes, and Elusimicrobia, as well as fungal Glomeromycota, were decreased by elevated ozone (*P < *0.05). These ozone-responsive phyla were generally correlated (*P < *0.05) with plant biomass, plant carbon (C) uptake, and soil dissolved organic C, demonstrating that elevated ozone affects plant-microbe interactions. Our study highlighted that microbial responses to elevated ozone display a phylogenetic clustering pattern, suggesting that response strategies to elevated ozone stress may be phylogenetically conserved ecological traits.

**IMPORTANCE** The interactions of plant and soil microbial communities support plant growth and health. The increasing tropospheric ozone decreases crop biomass and also alters soil microbial communities, but the ways in which crops and their associated soil microbial communities respond to elevated tropospheric ozone are not clear, and it is also obscure whether the interactions between ozone and the commonly applied *N* fertilization exist. We showed that the microbial responses to both elevated ozone and *N* fertilization were phylogenetically conserved. However, the microbial communities that responded to *N* fertilization and elevated ozone were different, and this was further verified by the lack of an interactive effect between *N* fertilization and elevated ozone. Given that the global tropospheric ozone concentration will continue to increase in the coming decades, the decrease of specific microbial populations caused by elevated ozone would result in the extinction of certain microbial taxa. This ozone-induced effect will further harm crop production, and awareness is urgently needed.

## INTRODUCTION

Although nitrogen (*N*) fertilization improves crop production (1), its overuse has resulted in excessive *N* accumulation in soil. In addition, the soil *N* input due to tropospheric *N* deposition is increasing globally, and it is projected to increase continuously within the first half of the 21^st^ century (2). The subsequent stimulation of soil microbial denitrification leads to the emission of nitrous oxide (N_2_O), a significant greenhouse gas (3). Nitrogen fertilization in agricultural soils also contributes to N_2_O emissions, representing a significant greenhouse gas (4, 5). Therefore, reducing *N* fertilization is necessary to mitigate global climate changes. Meanwhile, tropospheric ozone has increased since the industrial revolution because of the increased levels of reactive *N* oxide radicals and reduced volatile organic compounds (6, 7). As an essential component of air pollution and greenhouse gas, many countries face elevated tropospheric ozone problems (8), with the maximum daily average ozone concentration reaching up to 70 ppb across China in 2018 (9) and increasing by 1 to 2% annually throughout the 21^st^ century (10, 11). Tropospheric ozone can inhibit crop growth, photosynthesis, and flowering (12, 13), which thereby reduces crop production (14). In accordance, elevated tropospheric ozone decreased maize yields by approximately 10% in the United States from 1980 to 2011, based on historical observations (14), and it reduced 6 to 8% of annual crop yields in China (15). The *N* use efficiency of crops was decreased by elevated tropospheric ozone through decreasing *N* uptake, which is one of the main factors affecting crop yields (9).

Both *N* fertilization and elevated tropospheric ozone may affect the soil microbial community (16), and these factors play critical roles in mediating the geochemical cycles of carbon (C), *N*, phosphorus (P), and sulfur (17) that support plant growth (18, 19). Long-term *N* fertilization can decrease soil microbial biomass and diversity (20) and can also increase the ratio of Gram-positive to Gram-negative bacteria (2). In contrast, elevated ozone can either decrease (12) or increase (21) the soil microbial diversity. The relative abundance of certain soil bacterial populations can be altered by elevated ozone. For example, the nitrifying bacteria and *N*-fixing bacteria that are affiliated with *Sphingomonadaceae*, *Rhizobiaceae*, and *Nitrospiraceae* were increased by elevated ozone in maize soils (11). In addition, soil nutrient availability and resource distribution were altered under elevated ozone due to alterations in the ratio of fungi to bacteria (22). A 5-year elevated ozone treatment reduced the soil organic C (from 5.6 to 17%) and *N* (from 8.2 to 27.8%) contents, which reduced the *N* fertilization efficiency of crops (23). However, our previous studies found no interactive effects of *N* fertilization and elevated ozone on maize biomass and production (9). Whether there are interactive effects of *N* fertilization and elevated ozone on soil microbial communities remains obscure.

A meta-analysis study suggested that microbial responses to *N* fertilization are phylogenetically conserved (24). That is, closely related microbial taxa respond more similarly to *N* fertilization than do microbial taxa that are distantly related. Microbial responses to other environmental changes, such as drought, extreme desiccation, rewetting, and snowpack decline, are also phylogenetically conserved (25–28). In contrast, it is unclear whether microbial responses to elevated ozone are also phylogenetically conserved. In this study, three *N* fertilization levels (60, 120, and 240 kg *N* ha^−1^yr^−1^) and two ozone levels (ambient and ambient + 60 ppb ozone) were employed to investigate the effects of *N* fertilization levels and elevated ozone on the soil microbial community structure during the whole growth cycle of maize. The aim of this study was to address the questions of whether interactions between *N* fertilization and elevated ozone alter the soil microbial community structure, whether the soil microbial responses to *N* fertilization and elevated ozone are phylogenetically conserved in maize agroecosystems, which microbial populations are involved in the responses to *N* fertilization and elevated ozone, and how the microbial responses to elevated ozone affect maize.

## RESULTS

### Plant and soil geochemical properties.

Both *N* fertilization and elevated ozone exhibited significant effects on most plant and soil geochemical properties (Table S1). Specifically, *N* fertilization increased plant biomass, plant *N* uptake, plant C uptake, and soil ammonium (NH_4_^+^) concentration, whereas a decrease was observed in the soil available phosphorus (AP) (*P < *0.05) (Table S2). In contrast, elevated ozone decreased plant biomass, plant C uptake, soil pH, dissolved organic carbon (DOC), and AP but increased soil NH_4_^+^ and nitrate (NO_3_^−^). No interactive effects of *N* fertilization and elevated ozone were observed on plant and soil geochemical properties, except for the soil total potassium (TK) and available potassium (AK) (*P < *0.05) (Table S1).

10.1128/msystems.00721-22.1 TABLE S1 Effect of *N* fertilization and elevated ozone on plant and soil geochemical properties. The interactive effect of *N* and ozone was determined via a two-way ANOVA. *N* × O represents the interaction of *N* fertilization and elevated ozone. Bold values represent P < 0.05, and *n* indicates the sample size. Download Table S1, DOCX file, 0.02 MB. Copyright © 2023 Yu et al. 2023 Yu et al. https://creativecommons.org/licenses/by/4.0/ This content is distributed under the terms of the Creative Commons Attribution 4.0 International license.

10.1128/msystems.00721-22.2 TABLE S2 Plant and soil geochemical properties under *N* fertilization and ozone treatments. Letters represent the differences between groups. The differences between groups were determined via an ANOVA, and the *P* values were adjusted by the FDR method. The significant difference level is *P < *0.05, and *n* indicates the sample size. Download Table S2, DOCX file, 0.02 MB. Copyright © 2023 Yu et al. 2023 Yu et al. https://creativecommons.org/licenses/by/4.0/ This content is distributed under the terms of the Creative Commons Attribution 4.0 International license.

### Microbial community composition and diversity.

**FIG 1:**
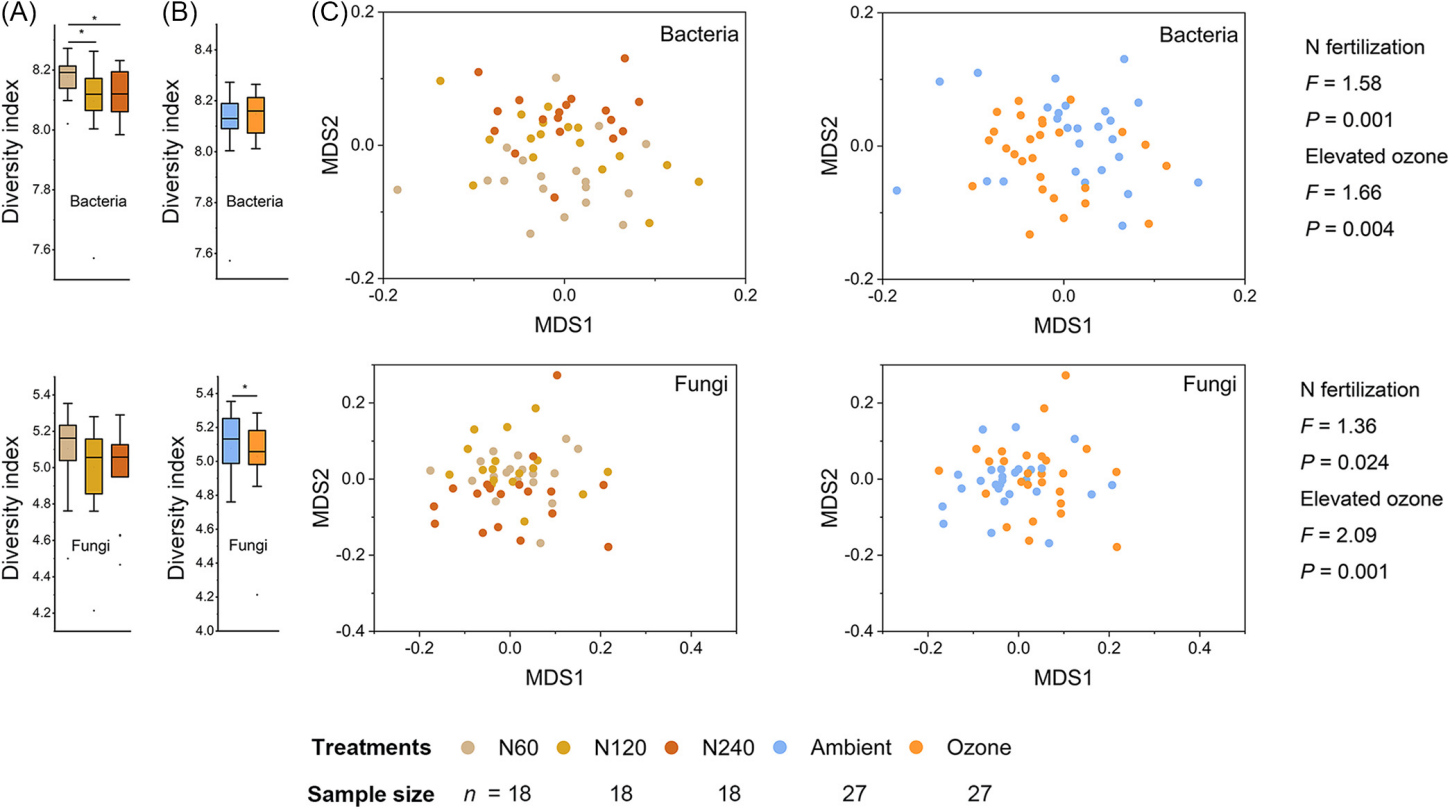
Influence of *N* fertilization and elevated ozone on microbial diversity. Alpha diversity of rhizospheric soil bacteria and fungi at the ASV level under *N* fertilization (A) and elevated ozone treatments (B). Individual boxes show the alpha diversity indices (Shannon-Winener) of samples under different treatments. Significance: *, *P* < 0.05. (C) Nonmetric multidimensional scaling based on the weighted Bray-Curtis dissimilarity via an Adonis analysis, showing the beta diversity of rhizospheric bacterial and fungal communities at the ASV level. Treatments are indicated by color, and the corresponding sample sizes are shown in the legend below. See “Statistical analyses” in Materials and Methods for more details on the statistical methods employed.

After resampling at 45,000 reads per sample, a total of 2,430,000 amplicon sequences for the 16S rRNA gene representing bacterial communities were generated, resulting in 18,260 amplicon sequence variants (ASVs). Most of these sequences were affiliated with *Proteobacteria* (24.8 to 26.3%), *Acidobacteria* (15.6 to 17.7%), and *Chloroflexi* (12.7 to 14.6%) (Fig. S1). The most abundant genera included *Anaerolineaceae* UTCFX1 (3.1 to 3.8%), *Nitrosomonadaceae* MND1 (1.8 to 2.5%), and *Pyrinomonadaceae* RB41 (1.9 to 2.3%) (Fig. S1B). The bacterial alpha diversity (Shannon index) was decreased (*P < *0.05) by *N* fertilization (Fig. 1A), but it was not affected by elevated ozone (Fig. 1B). No interactive effect between *N* fertilization and elevated ozone was observed on the bacterial alpha diversity (Table S3).

10.1128/msystems.00721-22.3 TABLE S3 Effect of *N* fertilization and elevated ozone on microbial diversity. The effect was determined via a two-way ANOVA. *N* × O represents the interaction of *N* fertilization and elevated ozone. Bold values represent *P < *0.05. Download Table S3, DOCX file, 0.01 MB. Copyright © 2023 Yu et al. 2023 Yu et al. https://creativecommons.org/licenses/by/4.0/ This content is distributed under the terms of the Creative Commons Attribution 4.0 International license.

10.1128/msystems.00721-22.7 FIG S1 Average relative abundance of bacterial and fungal composition. (A) Bacterial phylum composition. (B) Bacterial genus composition. (C) Fungal phylum composition. (D) Fungal genus composition. Treatments included two ozone levels (*A* initial, Ambient; *E* initial, Elevated O_3_) and three *N* fertilization levels (N60, N120, and N240) with 9 replicates. The relative abundances of the bacterial/fungal populations were above 1% in at least one sample. All of the relative abundances that were less than 1% were defined as others. Download FIG S1, TIF file, 2.8 MB. Copyright © 2023 Yu et al. 2023 Yu et al. https://creativecommons.org/licenses/by/4.0/ This content is distributed under the terms of the Creative Commons Attribution 4.0 International license.

Similarly, a total of 1,684,152 internal transcribed spacer (ITS) sequences were generated after resampling at 31,188 reads per sample, resulting in 4,036 ASVs representing the fungal communities. Most sequences were affiliated with *Ascomycota* (42.3 to 48.8%), *Glomeromycota* (9.6 to 16.8%), and *Basidiomycota* (3.6 to 6.4%) (Fig. S1C). The most abundant genera included *Gibberella* (6.0 to 9.4%), *Claroideoglomus* (4.1 to 6.4%), and Fusarium (4.0 to 5.0%) (Fig. S1D). Contrary to the results observed with the bacteria, the fungal alpha diversity was not affected by *N* fertilization (Fig. 1A) but was decreased (*P < *0.05) by elevated ozone (Fig. 1B). No interactive effect between *N* fertilization and elevated ozone was observed on the fungal alpha diversity (Table S3).

There were significant (Adonis, *P < *0.05) effects of *N* fertilization and elevated ozone on both the bacterial and fungal communities (Table S4). The effects of *N* fertilization (*F *= 1.58) and elevated ozone (*F *= 1.66) on the bacterial community were generally equal, but the effect of elevated ozone on the fungal community (*F *= 2.09) was much larger than that of *N* fertilization on the fungal community (*F *= 1.36) (Fig. 1C). There was no effect on the microbial communities due to an interaction between *N* fertilization and elevated ozone (*P > *0.1) (Table S4).

10.1128/msystems.00721-22.4 TABLE S4 Effect of *N* fertilization and elevated ozone on microbial community structure. The effect was determined via an Adonis analysis, based on the abundance weighted Bray-Curtis distances and the abundance unweighted Sorensen distances. *N* × O represents the interaction of *N* fertilization and elevated ozone. Bold values represent *P < *0.05. Download Table S4, DOCX file, 0.02 MB. Copyright © 2023 Yu et al. 2023 Yu et al. https://creativecommons.org/licenses/by/4.0/ This content is distributed under the terms of the Creative Commons Attribution 4.0 International license.

### Phylogenetic conservation of microbial responses to *N* fertilization.

**FIG 2:**
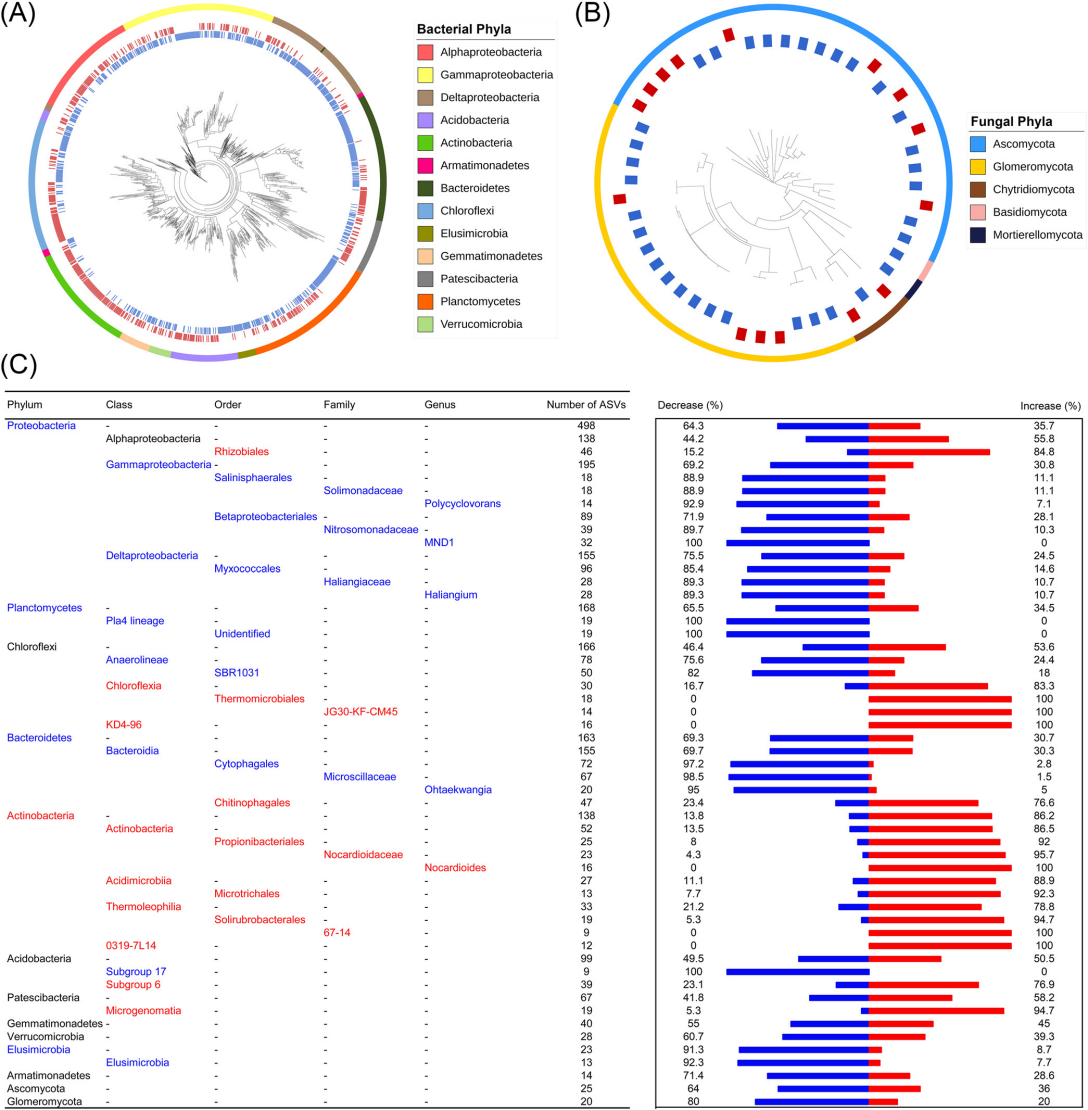
Phylogenetic distribution of the ASVs changed by *N* fertilization. Phylogenetic tree of the bacterial ASVs (A) and fungal ASVs (B) that changed by N fertilization. Taxonomic levels of the microbial response to *N* fertilization (C). The responses that were significantly more positive (red), more negative (blue), or displayed no significance (black) compared to that expected via random chance (two-tailed exact test; *P < *0.05) are shown. Only the phyla containing >10 bacterial ASVs are shown. The percentage of ASVs that were increased (red) or decreased (blue) by *N* fertilization are plotted in the bar graph to the right. Higher taxonomic levels are listed in black (e.g., the class Alphaproteobacteria) when only the lower levels are significant.

A total of 1,593 bacterial ASVs were changed in relative abundance by *N* fertilization (*P < *0.05) (Fig. 2A). These ASVs were mainly affiliated with *Proteobacteria* (31.3%), *Planctomycetes* (10.5%), *Chloroflexi* (10.4%), and *Bacteroidetes* (10.2%). 26.0% of the significantly changed ASVs were increased in relative abundance by at least 2-fold by *N* fertilization, and 45.3% of the significantly changed ASVs were decreased by at least 50% by *N* fertilization (Fig. S2A). 86.2% of the ASVs that were affiliated with *Actinobacteria* were increased by *N* fertilization (two-tailed exact test, *P < *0.05) (Fig. 2C). On the contrary, most of the ASVs affiliated with *Gammaproteobacteria* (69.2%), Deltaproteobacteria (75.5%), *Bacteroidetes* (69.3%), *Elusimicrobia* (91.3%), and *Planctomycetes* (65.5%) were decreased by *N* fertilization, suggesting that the bacterial response to *N* fertilization was largely at the phylum level. We also examined other taxonomic levels and found a consistent response to *N* fertilization (Fig. 2C). The mean genetic depth (τD) of ASVs with both positive and negative responses ranged from 0.041 to 0.049 (average τD = 0.045; permutation test, *P < *0.05) (Table 1), demonstrating an average sequence dissimilarity of approximately 9% in the 16S rRNA gene amplicon showing a consistent response to *N* fertilization.

**TABLE 1:** Mean genetic depth (τD) of consensus clades as calculated with the consenTRAIT algorithm

Treatments	Bacteria		Fungi	
	Positive response	Negative response	Positive response	Negative response
*N* fertilization	**0.041** ^*a*^	**0.049**	0.101	0.135
Elevated ozone	**0.051**	**0.052**	**0.182**	**0.255**

a Bold indicates that the response is significantly associated with phylogeny (permutation test; *P* < 0.05). Consensus clades are phylogenetic clades in which >90% of the descendant ASVs show the same direction of response.

10.1128/msystems.00721-22.8 FIG S2 Fold change and percentages of positive or negative response of microbial ASVs to *N* fertilization (A) and elevated ozone (B). The responses that were significantly more positive or negative than that expected by random chance (two-tailed exact test; *P < *0.05) are shown. Only the numbers of bacterial ASVs that were greater than 10 were used. Download FIG S2, TIF file, 2.7 MB. Copyright © 2023 Yu et al. 2023 Yu et al. https://creativecommons.org/licenses/by/4.0/ This content is distributed under the terms of the Creative Commons Attribution 4.0 International license.

A total of 96 fungal ASVs were changed in relative abundance by *N* fertilization, and these were mainly affiliated with *Ascomycota* (26.0%) and *Glomeromycota* (20.8%) (Fig. 2B). Even though 64% (16 out of 25 ASVs) of the ASVs affiliated with *Ascomycota* and 80% (16 out of 20 ASVs) of the ASVs affiliated with *Glomeromycota* were decreased by *N* fertilization (Fig. S2A), the changes were not significant (two-tailed exact test, *P > *0.05) (Fig. 2C). Besides, the τD of the ASVs with both positive and negative responses were not significant (permutation test, *P > *0.05) (Table 1), indicating that the fungal responses to *N* fertilization were not phylogenetically conserved.

### Phylogenetic conservation of microbial responses to elevated ozone.

**FIG 3:**
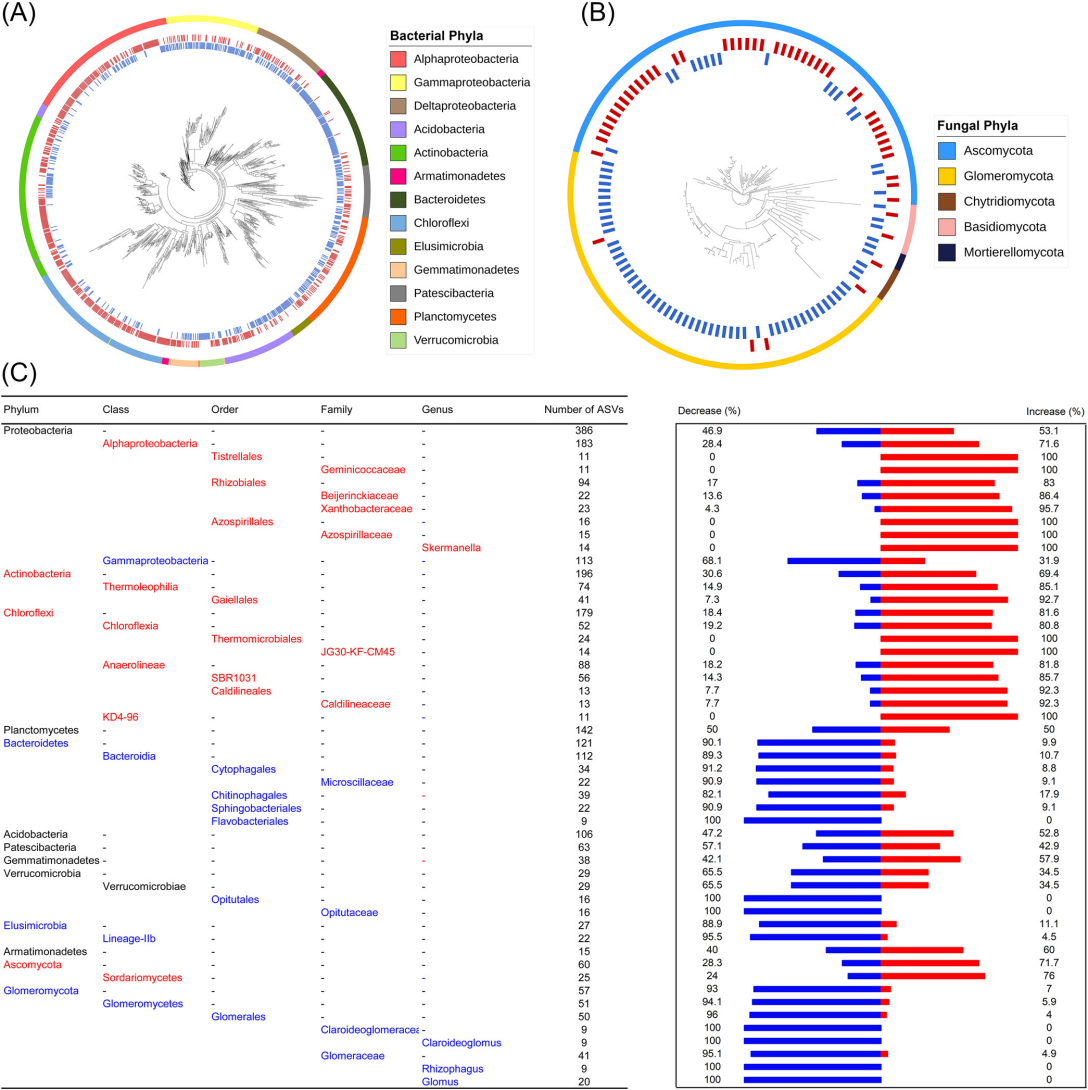
Phylogenetic distribution of the ASVs changed by elevated ozone. Phylogenetic tree of the bacterial ASVs (A) and fungal ASVs (B) that changed by elevated ozone. Taxonomic levels of the microbial response to elevated ozone (C). The responses that were significantly more positive (red), more negative (blue), or displayed no significance (black) compared to that expected via random chance (two-tailed exact test; *P < *0.05) are shown. Only the phyla containing >10 bacterial ASVs are shown. The percentage of ASVs that were increased (red) or decreased (blue) by elevated ozone are plotted in the bar graph to the right. Higher taxonomic levels are listed in black (e.g., the class Verrucomicrobiae) when only the lower levels are significant.

A total of 1,387 bacterial ASVs were changed by elevated ozone (Fig. 3A). 25.6% (355 out of 1,387 ASVs) of these ASVs were increased in relative abundance by at least 2-fold by elevated ozone, and 29.0% (402 out of 1,387 ASVs) of these ASVs were decreased by at least 50% by elevated ozone. Most of the ASVs affiliated with *Alphaproteobacteria* (71.6%), *Actinobacteria* (69.4%), and *Chloroflexi* (81.6%) were increased, whereas most of the ASVs affiliated with *Gammaproteobacteria* (68.1%), *Bacteroidetes* (90.1%), and *Elusimicrobia* (88.9%) were decreased by elevated ozone (two-tailed exact test, *P < *0.05) (Fig. S2B). This suggests that the bacterial responses to elevated ozone occur at the phylum level. We also examined other taxonomic levels and found a consistent response to elevated ozone (Fig. 3C). The mean τD of ASVs with positive and negative responses ranged from 0.050 to 0.052 (average τD = 0.051; permutation test, *P < *0.05) (Table 1), demonstrating an average sequence dissimilarity of approximately 10.2% in the 16S rRNA gene amplicon showing a consistent response to elevated ozone.

A total of 220 fungal ASVs were significantly changed by elevated ozone (Fig. 3B). Among them, 33.6% of these ASVs were increased in relative abundance by at least 2-fold by elevated ozone, and 45.9% of these ASVs were decreased by at least 50% (Fig. S2B). These 220 fungal ASVs were mainly affiliated with *Ascomycota* (27.3%) and *Glomeromycota* (25.9%). 71.7% (43 out of 60 ASVs) of the ASVs affiliated with *Ascomycota* were increased, whereas 93.0% (53 out of 57 ASVs) of the ASVs affiliated with *Glomeromycota* were decreased by elevated ozone (two-tailed exact test, *P < *0.05) (Fig. S2B). All taxonomic levels showed consistent responses to elevated ozone (Fig. 3C). The mean τD of the ASVs with positive and negative responses ranged from 0.18 to 0.26 (average τD = 0.22; permutation test, *P < *0.05) (Table 1).

### Comparison of microbial responses to *N* fertilization and elevated ozone.

The phyla *Proteobacteria*, *Actinobacteria*, and *Bacteroidetes* were significantly changed by both *N* fertilization and elevated ozone (Fig. 2 and 3). However, only 3% of bacterial ASVs and 2% of fungal ASVs overlapped in response to *N* fertilization and elevated ozone (Fig. S3), suggesting that *N* fertilization and elevated ozone changed different microbial populations. The average τD of the bacterial response to *N* fertilization was 0.045, suggesting that the bacterial response to *N* fertilization was between the family and genus levels (Fig. S4). However, the average τD of the bacterial response to elevated ozone was 0.051, suggesting that the bacterial response to *N* fertilization was between the family and order levels. Hence, the bacterial response to elevated ozone was slightly more conserved than was the bacterial response to *N* fertilization.

10.1128/msystems.00721-22.9 FIG S3 Venn diagram of the ASVs shared between the *N* -responsive community and the ozone-responsive community. Download FIG S3, TIF file, 2.8 MB. Copyright © 2023 Yu et al. 2023 Yu et al. https://creativecommons.org/licenses/by/4.0/ This content is distributed under the terms of the Creative Commons Attribution 4.0 International license.

10.1128/msystems.00721-22.10 FIG S4 Phylogenetic depths of the bacterial responses to *N* fertilization and elevated ozone. For comparison, rough taxonomic levels are shown on the top axis. Download FIG S4, TIF file, 1.8 MB. Copyright © 2023 Yu et al. 2023 Yu et al. https://creativecommons.org/licenses/by/4.0/ This content is distributed under the terms of the Creative Commons Attribution 4.0 International license.

### Relationships of the significantly changed microbial populations with plant and soil geochemical properties.

Plant biomass, plant *N* uptake, and plant C uptake were positively correlated with *Actinobacteria*, which were increased by *N* fertilization (*P < *0.05) (Table S5). In contrast, these properties were negatively correlated with *Gammaproteobacteria*, Deltaproteobacteria, *Bacteroidetes*, *Elusimicrobia*, and *Planctomycetes*, all of which were decreased by *N* fertilization.

10.1128/msystems.00721-22.5 TABLE S5 Pearson correlations of plant and soil geochemical properties with ASVs responding consistently to *N* fertilization at the phylum level. Bold values represent *P < *0.05 after being adjusted by the FDR method. Download Table S5, DOCX file, 0.02 MB. Copyright © 2023 Yu et al. 2023 Yu et al. https://creativecommons.org/licenses/by/4.0/ This content is distributed under the terms of the Creative Commons Attribution 4.0 International license.

**FIG 4:**
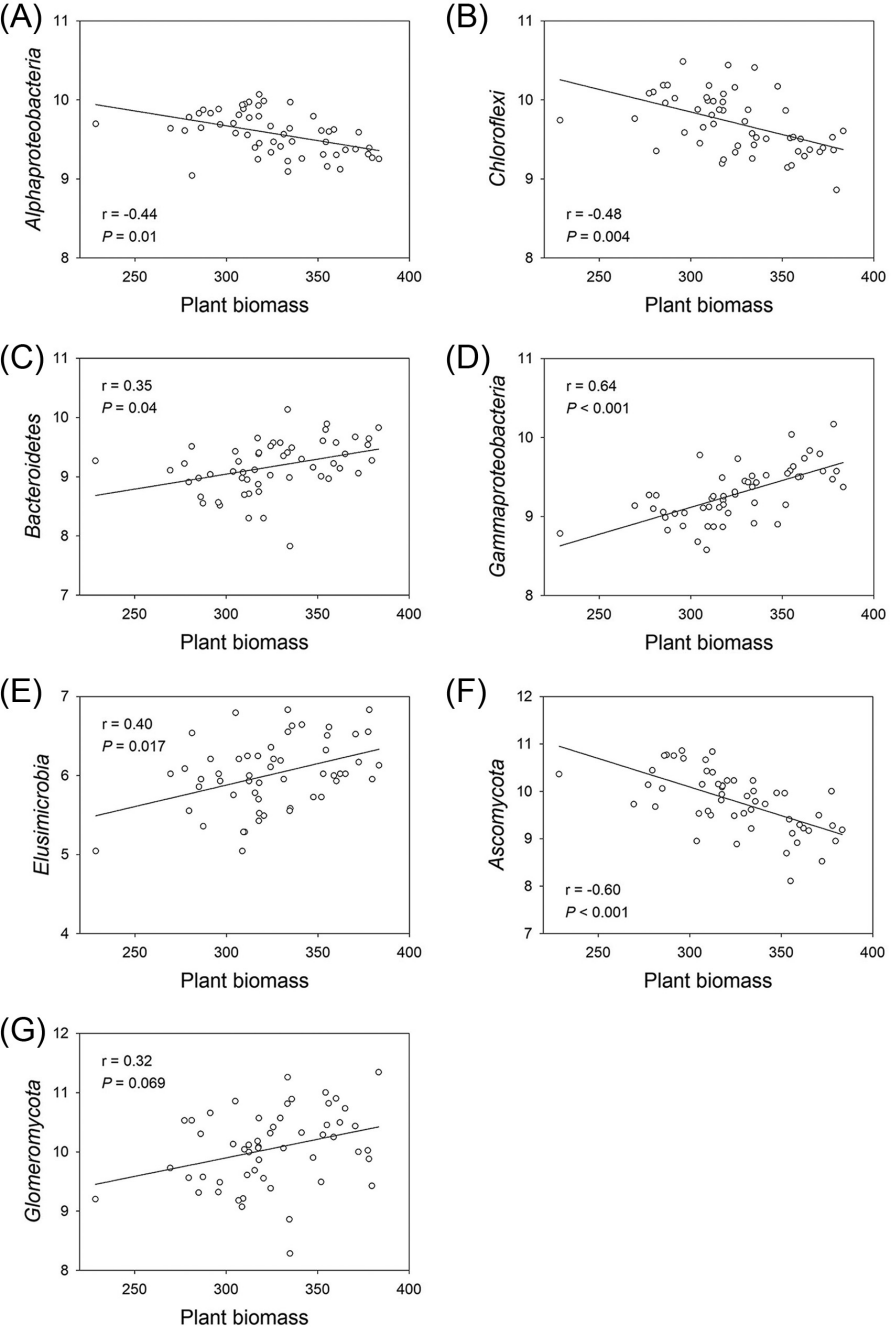
Elevated ozone modulated the relationships between microbial composition and plant biomass. Relationships between the relative abundance of ozone-responsive bacterial Alphaproteobacteria, Chloroflexi, Bacteroidetes, Gammaproteobacteria, and Elusimicrobia (A–E), as well as fungal Ascomycota and Glomeromycota (F and G) with plant biomass. Relationships were considered significant if *P < *0.05.

Plant biomass was also negatively correlated with the phyla *Alphaproteobacteria* and *Chloroflexi* (*P < *0.05) (Fig. 4; Table S6), both of which were increased by elevated ozone. However, plant biomass was positively correlated with all of the phyla that showed negative responses to elevated ozone. The soil DOC was positively correlated with the bacterial phyla that showed positive responses to elevated ozone, except for *Alphaproteobacteria*. For the fungal communities, plant biomass and plant C uptake were marginally and positively (*P < *0.1) correlated, and the soil DOC was significantly and positively (*P < *0.05) correlated with *Glomeromycota*, which showed a negative response to elevated ozone. Plant biomass and plant C uptake were negatively correlated (*P < *0.05) with *Ascomycota*, which positively responded to elevated ozone.

10.1128/msystems.00721-22.6 TABLE S6 Pearson correlations of plant and soil geochemical properties with ASVs responding consistently to elevated ozone at the phylum level. Bold values represent *P < *0.05 after being adjusted by the FDR method. Download Table S6, DOCX file, 0.02 MB. Copyright © 2023 Yu et al. 2023 Yu et al. https://creativecommons.org/licenses/by/4.0/ This content is distributed under the terms of the Creative Commons Attribution 4.0 International license.

## DISCUSSION

Long-term *N* fertilization usually decreases both bacterial and fungal alpha diversity (29, 30), especially under high *N* fertilization levels (29). In this study, *N* fertilization also decreased (*P < *0.05) the bacterial alpha diversity (Fig. 1). In contrast, no significant effect of *N* fertilization on fungal alpha diversity was observed, despite a decreasing trend of fungal alpha diversity with increasing *N* fertilization levels. This may be due to the shorter duration of *N* fertilization in this study than in other studies (30). Elevated ozone decreased (*P < *0.05) the fungal alpha diversity, which was also found in the soils of two endemic trees in subtropical China (31). No interactive effects of *N* fertilization and elevated ozone were observed on plants, and most soil geochemical properties closely correlated with the microbial community (32), indicating that *N* fertilization could not alleviate the adverse effect of elevated ozone on the plant and microbial communities. Together, our findings revealed no interactive effect of *N* fertilization and elevated ozone at the tested levels on the soil microbial community.

Microbial responses to environmental changes, including drought, specific carbon resource utilization, precipitation, and *N* fertilization can be phylogenetically conserved (24, 26, 27, 33). In this study, bacteria with positive and negative responses to *N* fertilization were phylogenetically conserved at genetic depths (τD) of 0.041 and 0.049, respectively (Table 1). A previous study showed that the positive response of bacteria to over 3 years of *N* addition was conserved at a genetic depth of 0.020 (27). A meta-analysis of soil bacterial communities from 13 field experiments across 5 continents was conducted. The results showed that the bacterial responses to *N* addition were phylogenetically conserved both within (τD = 0.018) and across (τD = 0.017) sites (24). These values are comparable to, albeit smaller than, our observed result (τD = 0.045), probably owing to the larger, more significant amount of *N* used in this study. The bacterial responses to elevated ozone were also phylogenetically conserved. The bacterial positive and negative responses to elevated ozone were phylogenetically conserved at genetic depths (τD) of 0.051 and 0.052, which are slightly higher than the conserved genetic depths in response to *N* fertilization. Similarly, the bacterial responses to drought and nitrogen fertilization were also conserved in similar phylogenetic depths, based on the field manipulation of precipitation and nitrogen fertilization for over 3 years (27).

The fungal community was more influenced by elevated ozone than by *N* fertilization (Fig. 1), with a more prominent τD value being observed in response to elevated ozone. These results indicated that the fungal responses to elevated ozone are more phylogenetically conserved than are those to *N* fertilization. In nutrient-rich agroecosystems, the fungal responses to ecological disturbances are more conserved, compared with cases without disturbance (resource utilization) (24), because the reactions to disturbances involve the multilocus of the whole-genome more so than do cases of resource utilization (34). While under *N* deficiency, fungi tend to allocate their resources to facilitate survival rather than to thrive (35). This indicates that fungal responses would be less phylogenetically conserved under nutrient-poor conditions than under lethal stress.

Nitrogen fertilization increased the relative abundance of Actinobacteria but decreased the relative abundances of Gammaproteobacteria, Deltaproteobacteria, Bacteroidetes, Elusimicrobia, and Planctomycetes (Fig. 2). The increase of Actinobacteria by *N* fertilization was consistent with the results of a previous study that showed that Actinobacteria was increased by *N* addition (24). The rise of Actinobacteria by *N* fertilization was highly correlated with the increase of plant biomass and soil NH_4_^+^. This is consistent with the results of a long-term *N* fertilization study that showed that *N* fertilization promoted the growth of Actinobacteria in agroecosystems and coincided with an increased soil available *N* by *N* fertilization (20). The phylogenetically conserved phyla showing negative responses to *N* fertilization were Gammaproteobacteria, Deltaproteobacteria, Bacteroidetes, Elusimicrobia, and Planctomycetes, and this result is also broadly consistent with the results of a previous study (24). Some bacterial populations that are affiliated with Proteobacteria and Planctomycetes are *N*-fixing populations (36) and are dispensable upon *N* fertilization.

Elevated ozone increased the relative abundances of Alphaproteobacteria, Actinobacteria, and Chloroflexi and decreased the relative abundances of Gammaproteobacteria, Bacteroidetes, and Elusimicrobia (Fig. 3). Considering that most of the populations that are affiliated with Bacteroidetes and Elusimicrobia are obligate anaerobes, their abundance might be strongly inhibited by oxygen radicals when under an elevated ozone level (37, 38). In the present study, Chloroflexi increased with elevated ozone (Fig. 3). This is in line with the results of a previous study, in which Chloroflexi was one of the dominant bacterial phyla after an ozone treatment in a bioreactor (39), suggesting the strong resistance of Chloroflexi to ozone. The increase in soil NO_3_^−^ induced by elevated ozone can be attributed to the increase in nitrite-oxidizing Chloroflexi (40), which thus favors the nitrate-reducing Chloroflexi (41). Actinobacteria were increased under elevated ozone. Such an increase of Actinobacteria was mainly correlated with the increases in soil NH_4_^+^ and NO_3_^−^, which provide more available *N* for the growth of Actinobacteria (20).

The only fungal response to elevated ozone was phylogenetically conserved (Table 1). Elevated ozone increased the relative abundance of Ascomycota but decreased the relative abundance of Glomeromycota (Fig. 3). Plant properties and the soil DOC showed significant correlations with the fungal populations that were changed by elevated ozone (Table S6). Ascomycota, one of the major components of plant-pathogenic fungi (42, 43), presented a negative correlation (*P < *0.001) with plant properties, suggesting a potential threat to plant health under increasing ozone. Glomeromycota, dominated by arbuscular mycorrhizal fungi (AMF), showed a negative response to elevated ozone. A recent study also found that elevated ozone changed the AMF community composition and decreased AMF colonization (44). The growth of Glomeromycota depends on the soil DOC and is also mainly derived from the root exudation of plants (45). A recent analysis, including 239 studies exploring the dry root masses of woody plants, found that elevated ozone generally decreased the root biomass (46). Hence, elevated ozone typically reduces the allocation of plant C resources to the soil, and this is consistent with the reduced soil DOC by elevated ozone that was found in this study (Table S2). Glomeromycota forms symbioses with the roots, contributes to plant growth and production (47, 48), and enhances the environmental adaption of the plant to complicated environments (49). The inoculation of AMF increased the shoot biomass by 68% and the root biomass by 131% when the ozone concentrations were over 80 ppb, and crop production was then increased under elevated ozone stress, based on a meta-analysis that included 20 studies (50). Therefore, the increase of Ascomycota and the decrease of Glomeromycota under elevated ozone may result in stunted growth and plant disease and may further decrease plant biomass and production. In summary, we provide evidence that the microbial responses to elevated ozone are phylogenetically conserved. As no interactive effect of *N* fertilization and elevated ozone was observed on microbial communities and plant and soil geochemical properties, the adverse effects of elevated ozone on plant and microbial communities could not be alleviated by *N* fertilization. The decrease of AMF that is induced by elevated ozone would aggravate the adverse effect on crops if no policy were proposed to impede the increase in tropospheric ozone. Furthermore, a decrease in specific microbial species may result in the extinction of microbial communities, warranting attention to the protection of microbial diversity and agriculture development from the damage of increasing tropospheric ozone.

## MATERIALS AND METHODS

### The study site and experimental manipulations.

The soils used in the present study were collected from an agricultural station located in Tangjiapu (40° 48’ *N*, 115° 99’ E), China (9). This station is located in the northwest of Beijing, which has a typical warm temperate and a semihumid continental climate. The meteorological conditions at this site are described in previous papers (9, 51). In brief, the annual mean temperature and precipitation from 2006 to 2016 were 9.9°C and 467 mm.

Maize is one of the major crops throughout the world, and it is grown mainly in the United States, Europe, China, and Argentina (52). Maize cultivar Zhengdan 958 was selected as the test crop, as it is the most commonly grown in China. Maize was planted in 6.1 L pots (the areas of the inner top circles were 615.7 cm^2^). The homogeneous soil mixture for maize growth contained an equal volume of planting soil (i.e., a mixture of recycled forest products and peat moss) and local sandy loam soil (taken at depths of 0 to 10 cm from the study site, air-dried, and sieved through a 3 mm mesh). The optimum *N* fertilization rate ranged from 123 to 268 kg *N* ha^−1^ in continuous maize and from 42 to 241 kg *N* ha^−1^ in the yearly soybean-maize rotation (1). Hence, 3 *N* fertilization levels, all of which are in the range of the optimum *N* fertilization rate, were randomly distributed in the open-top growth chambers (OTCs): N60, 60 kg *N* ha^−1^yr^−1^; N120, 120 kg *N* ha^−1^yr^−1^; and N240, 240 kg *N* ha^−1^yr^−1^. The maximum daily average ozone concentration would reach up to 70 ppb in China, and the concentration of tropospheric ozone ranged from 30 ppb to 70 ppb when we conducted this experiment (9). So, we chose an elevated ozone concentration of 60 ppb, which is double the amount of the average ozone concentration and will be faced in the latter part of the 21^st^ century. After the plants reached the 4-leaf stage, elevated ozone treatments were performed on June 30, 2019, to elevate the tropospheric ozone concentration by 60 ppb. the elevated ozone treatments were applied using an electrical discharge ozone generator (HY003, Chuangchen Co., Jinan, China) whose concentrations were monitored by an UV absorption ozone analyzer (Model 49i; Thermo Scientific, Franklin, MA, USA). OTCs supplied with ambient air were used as controls. Six OTCs made with toughened glass were used in this study, three of which were used for the elevated ozone treatments. In addition, three replicates for each *N* fertilization treatment were distributed in each OTC. In total, six treatments and nine replicates for each treatment in this study were conducted until plant maturity (September 24, 2019).

### Sample collection.

Maize plants and rhizospheric soil samples were collected at the end of September of 2019. Plants from each pot were excavated using a shovel, and the bulk soil (i.e., soil loosely attached to roots) was removed. Approximately 30 maize root pieces (10 cm from the root tip) were sampled from each pot and were stored in sealed polypropylene bags at 4°C during transportation. Rhizospheric soil (i.e., soil tightly adhering to roots) was collected using tweezers and was divided into two subsamples. The subsamples to be used in 16S rRNA amplicon sequencing (*n* = 9) were immediately frozen with liquid nitrogen and stored in RNase-free tubes at −80°C. The subsamples to be used for soil geochemical analyses (*n* = 9) were sieved (2 mm) and air-dried at room temperature. The soil ammonium and nitrate levels were analyzed immediately after the samples arrived at the laboratory (within 1 day).

### Determination of plant properties and soil geochemical properties.

The soil pH was measured using a pH meter (Model PHS-3C, Shanghai Precision and Scientific Instrument Co. Ltd., Shanghai, China) after shaking the soil in deionized water (1:2.5 wt/vol) suspensions for 30 min. The soil total organic carbon (TOC) was determined via the potassium dichromate external heating method (53). The dissolved organic carbon (DOC) was extracted by adding 120 mL of deionized water to 40 g soil samples (1:3 wt/vol) as described (54). After being centrifuged at 4,000 rpm for 10 min and passed through a 0.45 μm membrane, the filtered extracts were used for the DOC analysis. The total *N* (TN) was determined via Kjeldahl digestion. Ammonium (NH_4_^+^) and nitrate (NO_3_^−^) were extracted by 0.01 M potassium chloride (1:10 wt/vol) for 30 min and were then detected by an auto-analyzer (Alpkem, Perstorp Analytical Company, Wilsonville, OR, USA). The content of the total phosphorus (TP) and the available phosphorus (AP) in the soil were determined via the molybdenum-antimony colorimetric method (55). The total potassium (TK) and available potassium (AK) were determined using an atomic absorption spectrophotometer (Z-2300, Hitachi, Japan). The plant biomass, plant carbon uptake, and plant *N* uptake were measured using an elemental analyzer (Vario EL III, Elementar, Germany) as previously described (9).

### Illumina sequencing of 16S rRNA genes and ITS amplicons.

The experiment included six treatments and nine biological replicates for each treatment. Thus, 6 × 9 = 54 soil samples (0.5 g each) were used in total to extract the total DNA, which was done using a PowerSoil Kit (MOBIO, Carlsbad, CA, USA). The bacterial 16S rRNA gene V4-V5 hypervariable region was amplified using the primers 515F (5′-GTGCCAGCMGCCGCGGTAA-3′) and 907R (5′-CCGTCAATTCCTTTGAGTTT-3′) combined with adapter sequences and barcode sequences. The ITS2 regions were amplified using the primers ITS3 (5′-GCATCGATGAAGAACGCAGC-3′) and ITS4 (5′-TCCTCCGCTTATTGATATGC-3′). The purified amplicons were sequenced by Magigene Inc., Guangzhou, China, on a HiSeq2500 platform (Illumina Inc., San Diego, CA, USA). Chimera detection and removal were accomplished using the Gold Chimera-Free reference database via the USEARCH option in the UCHIME algorithm. The quality-filtered reads were truncated to an equal length. Unosie3 was applied to generate the ASV tables, and the ASVs were calculated using the usearch -unoise3 command. The representative sequence of each ASV was assigned to a taxonomic lineage and classified against the SILVA database (version 132) for the 16S rRNA gene or the UNITE database (version 7.2) for the ITS sequences.

### Statistical analyses.

An analysis of variance (ANOVA) was applied to test the effects of *N* fertilization and elevated ozone on plant and soil geochemical properties and the microbial diversity. The false discovery rate (FDR) correction was performed to adjust the obtained *P*-values for multiple comparisons.

The alpha diversity indices were calculated using the R functions “alpha.g” in the “vegan” and “ieggr” packages (56). A nonmetric multidimensional scaling (NMDS) analysis based on the abundance-weighted Bray-Curtis distance was used to compare the microbial communities in the different treatments. A permutational multivariate analysis of variance (Adonis) based on the Bray-Curtis distance was used to determine the microbial differences between treatments.

The significance of the ASVs that were changed by elevated ozone or *N* fertilization was determined using Student’s *t* test. For the *N* fertilization treatments, the significantly changed ASVs were those first selected with *P < *0.05 between N240 and N60. Then, the ASVs that increased with the *N* fertilization levels and the ASVs that decreased with the *N* fertilization levels were selected, respectively. For the ozone treatments, the significantly changed ASVs were those selected with *P < *0.05 between ambient and elevated ozone treatments. The ASVs that were increased by *N* fertilization or elevated ozone treatments were defined as positive responses. The ASVs that were decreased by *N* fertilization or elevated ozone treatments were defined as negative responses. A Pearson correlation analysis was conducted to evaluate the correlations between the significantly changed microbial populations and the plant and soil geochemical properties. The FDR correction was performed to adjust the *P*-values.

To assess whether the microbial responses to *N* fertilization or elevated ozone are phylogenetically conserved, representative sequences of these significantly changed ASVs were aligned using the DECIPHER package (57). An ML tree with 100 bootstrap replications was constructed with RAxML v8.0, using the GTR + Gamma distribution model (58). We then applied a consenTRAIT analysis to test whether the response of an ASV to *N* fertilization or elevated ozone was related to the microbial phylogeny (33). The tree was traversed from the root to the tips, recording the deepest nodes at which >90% of the descending tips (ASVs) shared the same directional response (a “consensus” clade). The genetic depth (average distance from the node to its descending tips) and the size of each consensus clade (total number of descending tips) were calculated. The genetic depths of clades with a single descending tip (i.e., ASV) were calculated as half of the branch length to the nearest neighbor, as previously recommended (33). Finally, the mean genetic depth, τD, of the consensus clades sharing positive or negative responses was calculated. Simulated τD values were calculated by randomizing the responses among the tips 1,000 times. This was done to assess the statistical significance of the phylogenetic conservation of the *N* fertilization levels or the elevated ozone. The probability of the phylogenetic conservation (nonrandomness) of the traits was calculated as the fraction of the simulated τD values that were greater than or equal to the observed τD (24).

The taxonomy of clades whose responses to *N* fertilization levels or elevated ozone were significantly more positive or negative than those that were expected by chance was calculated, based on the number of ASVs that had a positive or negative response at each taxonomic level. We performed a two-tailed Fisher’s exact test against the equal distribution of positive and negative responses at each taxonomic level.

### Data availability.

The raw sequence reads of the 16S rRNA gene and the ITS amplicons were deposited to the National Center for Biotechnology Information (NCBI) Sequence Read Archive (SRA) under BioProject ID PRJNA791240. The R scripts that were used are publicly available at https://github.com/yuzs8911/ozone_maize.
