# Supplementary material for: Phylogenetic Conservation of Soil Microbial Responses to Elevated Tropospheric Ozone and Nitrogen Fertilization
Source: mSystems. 2023 Jan 10;8(1):e00721-22. doi: 10.1128/msystems.00721-22 (PMC9948724; doi:10.1128/msystems.00721-22)
Supplement: TABLE S1 [file msystems.00721-22-s0007.docx]

|  | N fertilization  (*n* = 27) | | Elevated ozone  (*n* = 18) | | N × O  (*n* = 54) | |
| --- | --- | --- | --- | --- | --- | --- |
|  | F | *P* | F | *P* | F | *P* |
| Plant biomass | 9.9 | **< 0.001** | 43.4 | **< 0.001** | 0.20 | 0.82 |
| Plant N uptake | 59.3 | **< 0.001** | 0.6 | 0.44 | 0.97 | 0.39 |
| Plant C uptake | 10.1 | **< 0.001** | 42.3 | **< 0.001** | 0.04 | 0.96 |
| pH | 0.3 | 0.72 | 10.8 | **0.002** | 0.73 | 0.49 |
| TOC | 1.4 | 0.27 | 0.0 | 0.87 | 0.33 | 0.72 |
| TN | 0.3 | 0.75 | 1.3 | 0.25 | 1.76 | 0.18 |
| TP | 0.5 | 0.62 | 2.3 | 0.14 | 0.22 | 0.80 |
| TK | 0.6 | 0.57 | 0.3 | 0.58 | 0.05 | **0.047** |
| DOC | 0.3 | 0.72 | 19.0 | **< 0.001** | 2.76 | 0.07 |
| NH_4_^+^ | 9.2 | **< 0.001** | 7.4 | **0.009** | 0.46 | 0.63 |
| NO_3_^-^ | 3.0 | 0.06 | 30.0 | **< 0.001** | 0.56 | 0.57 |
| AP | 13.9 | **< 0.001** | 10.2 | **0.003** | 0.56 | 0.58 |
| AK | 0.1 | 0.87 | 0.6 | 0.81 | 4.90 | **0.01** |
