# Supplementary material for: Phylogenetic Conservation of Soil Microbial Responses to Elevated Tropospheric Ozone and Nitrogen Fertilization
Source: mSystems. 2023 Jan 10;8(1):e00721-22. doi: 10.1128/msystems.00721-22 (PMC9948724; doi:10.1128/msystems.00721-22)
Supplement: TABLE S2 [file msystems.00721-22-s0006.docx]

|  | N60  (*n* = 18) | N120  (*n* = 18) | N240  (*n* = 18) | Ambient ozone  (*n* = 27) | Elevated ozone  (*n* = 27) |
| --- | --- | --- | --- | --- | --- |
| Plant biomass (g/plant) | 309.4±27.5b | 323.5±37.9ab | 342.6±22.5a | 345.2±25.8a | 305.1±25.5b |
| Plant N uptake (g/plant) | 1.7±0.2c | 1.9±0.2b | 2.4±0.2a | 2.0±0.3a | 2.0±0.4a |
| Plant C uptake (g/plant) | 134.1±11.8b | 139.9±15.6ab | 148.3±10.3a | 149.2±10.8a | 132.3±111b |
| pH | 7.9±0.09a | 8.0±0.06a | 7.9±0.05a | 8.0±0.06a | 7.9±0.07b |
| TOC (g/kg) | 67.1±4.4a | 65.1±7.7a | 68.3±4.8a | 66.7±4.9a | 67.0±6.8a |
| TN (g/kg) | 2.6±0.3a | 2.5±0.4a | 2.5±0.2a | 2.6±0.3a | 2.5±0.2a |
| TP (g/kg) | 0.78±0.01a | 0.79±0.02a | 0.78±0.02a | 0.79±0.02a | 0.78±0.02a |
| TK (g/kg) | 16.0±0.4a | 16.1±0.4a | 15.9±0.7a | 16.1±0.6a | 16.0±0.5a |
| DOC (mg/kg) | 79.1±8.9a | 77.5±15.1a | 76.3±11.2a | 83.6±10.5a | 71.6±10.0b |
| NH_4_^+^ (mg/kg) | 5.5±0.9b | 7.0±1.8a | 7.2±1.2a | 6.1±1.7b | 7.1±1.3a |
| NO_3_^-^ (mg/kg) | 3.5±1.3a | 4.4±1.7a | 4.4±1.7a | 3.2±0.7b | 5.0±1.7a |
| AP (mg/kg) | 10.6±1.5a | 10.6±1.9a | 8.1±1.8b | 10.5±1.6a | 9.1±2.3b |
| AK (mg/kg) | 83.3±4.3a | 84.0±5.0a | 83.3±4.3a | 83.7±3.8a | 83.4±5.2a |
