# Supplementary material for: Phylogenetic Conservation of Soil Microbial Responses to Elevated Tropospheric Ozone and Nitrogen Fertilization
Source: mSystems. 2023 Jan 10;8(1):e00721-22. doi: 10.1128/msystems.00721-22 (PMC9948724; doi:10.1128/msystems.00721-22)
Supplement: TABLE S3 [file msystems.00721-22-s0005.docx]

|  | Bacteria | | | | Fungi | | |
| --- | --- | --- | --- | --- | --- | --- | --- |
|  | N fertilization | Elevated ozone | N × O | N fertilization | | Elevated ozone | N × O |
| Richness | 0.06 | 0.37 | 0.28 | **0.03** | | 0.82 | 0.17 |
| Shannon | 0.06 | 0.24 | 0.12 | 0.20 | | **0.05** | 0.15 |
