# Supplementary figures and images for: Phylogenetic Conservation of Soil Microbial Responses to Elevated Tropospheric Ozone and Nitrogen Fertilization
Source: mSystems. 2023 Jan 10;8(1):e00721-22. doi: 10.1128/msystems.00721-22 (PMC9948724; doi:10.1128/msystems.00721-22)

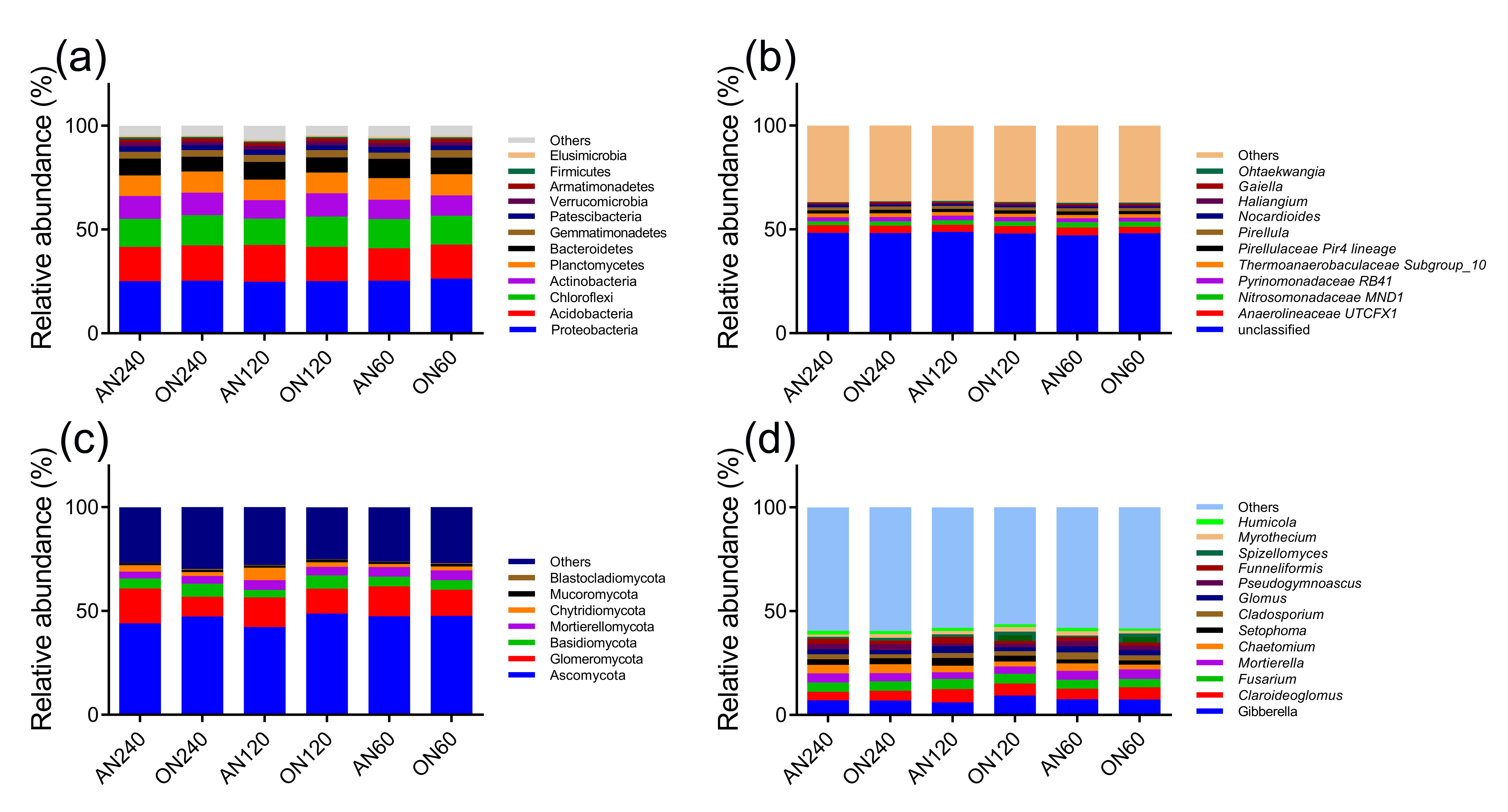

Supplement: FIG S1 [file msystems.00721-22-s0001.tif]

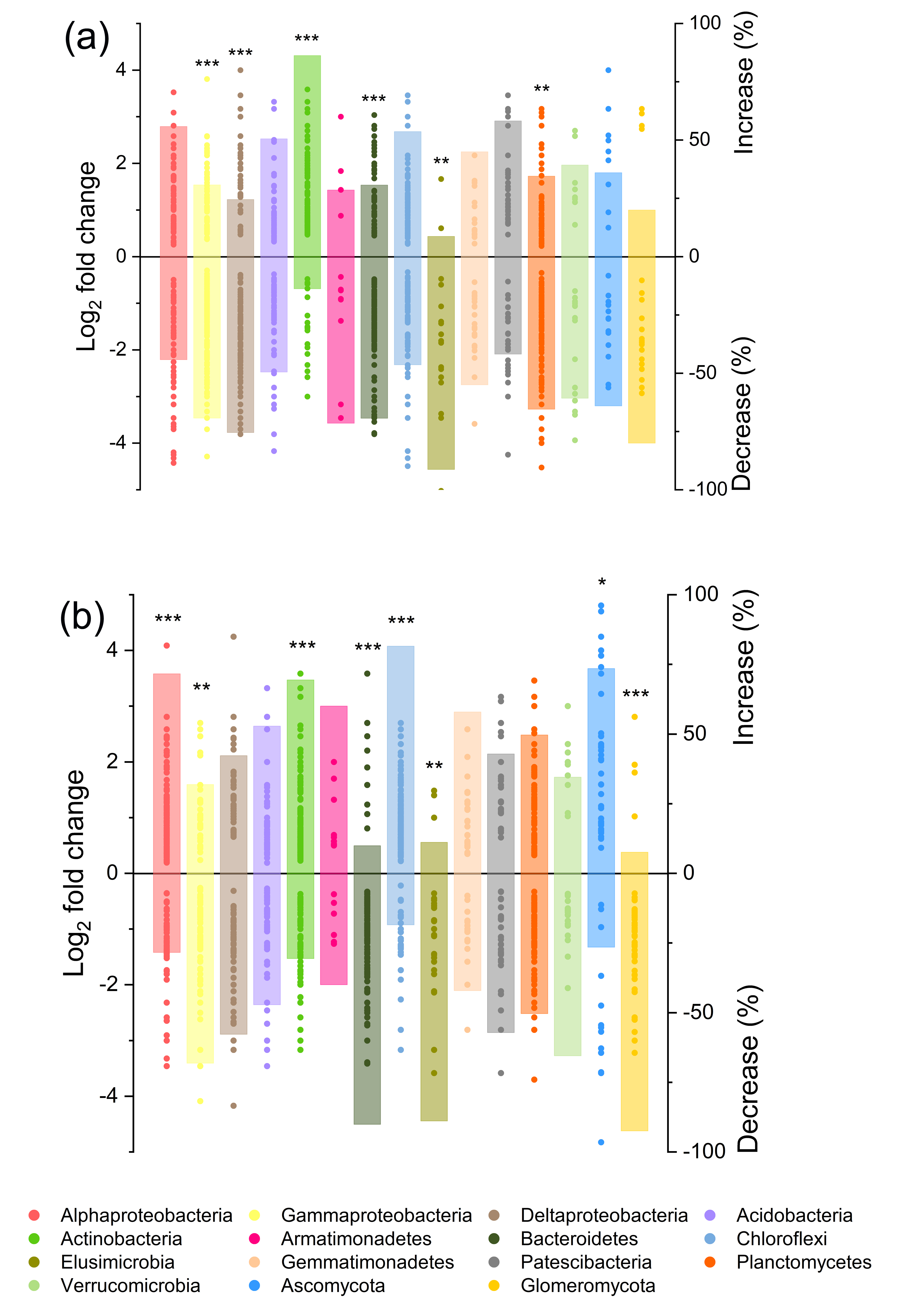

Supplement: FIG S2 [file msystems.00721-22-s0009.tif]

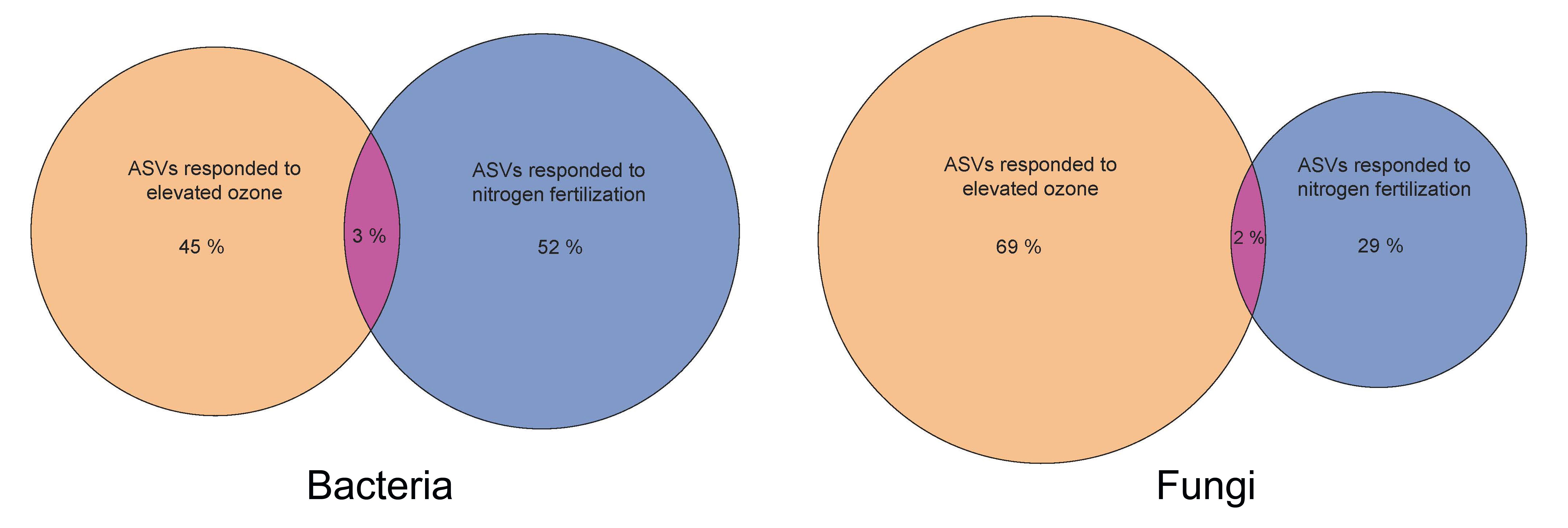

Supplement: FIG S3 [file msystems.00721-22-s0010.tif]

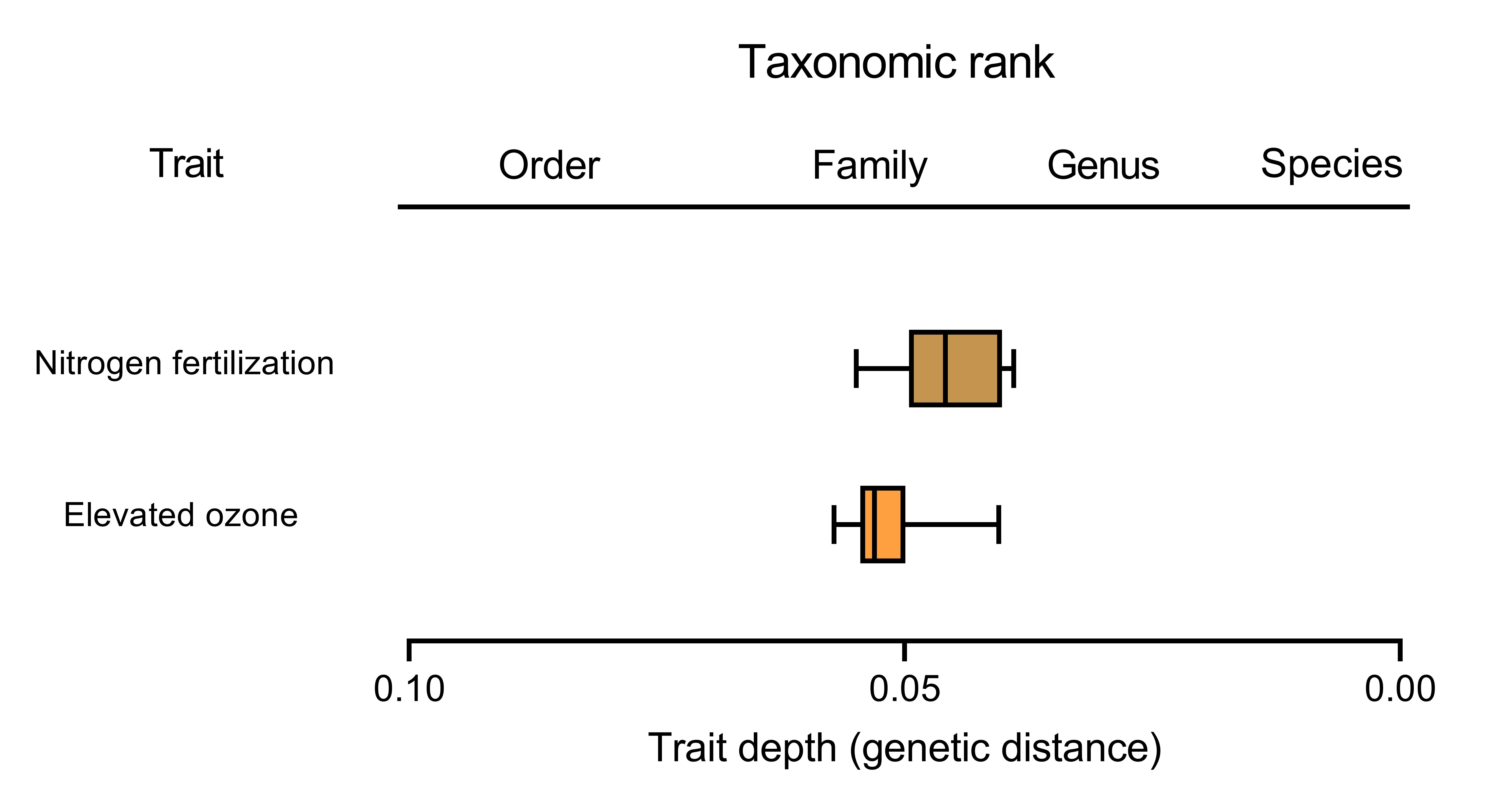

Supplement: FIG S4 [file msystems.00721-22-s0002.tif]
