# Supplementary material for: Phylogenetic Conservation of Soil Microbial Responses to Elevated Tropospheric Ozone and Nitrogen Fertilization
Source: mSystems. 2023 Jan 10;8(1):e00721-22. doi: 10.1128/msystems.00721-22 (PMC9948724; doi:10.1128/msystems.00721-22)
Supplement: TABLE S4 [file msystems.00721-22-s0004.docx]

|  | | Bray-Curtis | | | Sorensen | | |
| --- | --- | --- | --- | --- | --- | --- | --- |
|  |  | F | R^2^ | *P* | F | R^2^ | *P* |
| Bacteria | N fertilization | 1.58 | 0.058 | **0.001** | 1.3 | 0.048 | **0.001** |
|  | Elevated ozone | 1.66 | 0.03 | **0.004** | 1.3 | 0.024 | **0.004** |
|  | N × O | 0.96 | 0.035 | 0.632 | 0.97 | 0.036 | 0.776 |
| Fungi | N fertilization | 1.36 | 0.05 | **0.024** | 1.16 | 0.043 | **0.018** |
|  | Elevated ozone | 2.09 | 0.038 | **0.001** | 1.68 | 0.031 | **0.001** |
|  | N × O | 0.99 | 0.036 | 0.446 | 1.07 | 0.039 | 0.15 |
