# Supplementary material for: Phylogenetic Conservation of Soil Microbial Responses to Elevated Tropospheric Ozone and Nitrogen Fertilization
Source: mSystems. 2023 Jan 10;8(1):e00721-22. doi: 10.1128/msystems.00721-22 (PMC9948724; doi:10.1128/msystems.00721-22)
Supplement: TABLE S5 [file msystems.00721-22-s0003.docx]

|  | *Actinobacteria* | | *Gammaproteobacteria* | | *Deltaproteobacteria* | |  | *Bacteroidetes* | | *Elusimicrobia* | | *Planctomycetes* | |
| --- | --- | --- | --- | --- | --- | --- | --- | --- | --- | --- | --- | --- | --- |
|  | r | *P* | r | *P* | r | *P* |  | r | *P* | r | *P* | r | *P* |
| Plant biomass | 0.54 | **<0.001** | -0.55 | **<0.001** | -0.59 | **<0.001** |  | -0.39 | **0.016** | -0.37 | **0.023** | -0.59 | **<0.001** |
| Plant N uptake | 0.67 | **<0.001** | -0.66 | **<0.001** | -0.70 | **<0.001** |  | -0.58 | **<0.001** | -0.57 | **<0.001** | -0.72 | **<0.001** |
| Plant C uptake | 0.56 | **<0.001** | -0.56 | **<0.001** | -0.60 | **<0.001** |  | -0.39 | **0.016** | -0.37 | **0.027** | -0.58 | **<0.001** |
| pH | -0.05 | 0.832 | 0.13 | 0.590 | 0.02 | 0.922 |  | 0.09 | 0.780 | 0.14 | 0.538 | -0.02 | 0.900 |
| TOC | 0.22 | 0.234 | -0.18 | 0.404 | -0.09 | 0.780 |  | 0.07 | 0.782 | -0.03 | 0.900 | 0.00 | 0.975 |
| TN | 0.06 | 0.808 | -0.03 | 0.900 | 0.03 | 0.900 |  | -0.03 | 0.900 | 0.01 | 0.952 | -0.04 | 0.900 |
| TP | 0.24 | 0.201 | -0.05 | 0.832 | 0.02 | 0.900 |  | 0.15 | 0.516 | 0.09 | 0.780 | 0.06 | 0.832 |
| TK | -0.12 | 0.644 | 0.07 | 0.782 | 0.15 | 0.517 |  | 0.26 | 0.134 | 0.16 | 0.479 | 0.17 | 0.440 |
| DOC | 0.25 | 0.164 | 0.03 | 0.900 | 0.10 | 0.780 |  | 0.21 | 0.295 | 0.36 | **0.029** | 0.08 | 0.782 |
| NH_4_^+^ | 0.29 | 0.098 | -0.31 | 0.071 | -0.26 | 0.134 |  | -0.22 | 0.234 | -0.27 | 0.134 | -0.29 | 0.098 |
| NO_3_^-^ | 0.14 | 0.553 | -0.09 | 0.782 | -0.07 | 0.782 |  | -0.08 | 0.782 | -0.17 | 0.461 | -0.06 | 0.832 |
| AP | -0.27 | 0.134 | 0.28 | 0.114 | 0.33 | 0.054 |  | 0.32 | 0.065 | 0.41 | **0.012** | 0.36 | **0.028** |
| AK | 0.08 | 0.782 | -0.05 | 0.833 | -0.06 | 0.808 |  | -0.11 | 0.683 | -0.08 | 0.782 | -0.11 | 0.685 |
