# Supplementary material for: Phylogenetic Conservation of Soil Microbial Responses to Elevated Tropospheric Ozone and Nitrogen Fertilization
Source: mSystems. 2023 Jan 10;8(1):e00721-22. doi: 10.1128/msystems.00721-22 (PMC9948724; doi:10.1128/msystems.00721-22)
Supplement: TABLE S6 [file msystems.00721-22-s0008.docx]

|  | *Alphaproteobacteria* | | *Actinobacteria* | | *Chloroflexi* | | *Gammaproteobacteria* | | *Bacteroidetes* | | *Elusimicrobia* | | *Ascomycota* | | *Glomeromycota* | |
| --- | --- | --- | --- | --- | --- | --- | --- | --- | --- | --- | --- | --- | --- | --- | --- | --- |
|  | r | *P* | r | *P* | r | *P* | r | *P* | r | *P* | r | *P* | r | *P* | r | *P* |
| Plant biomass | -0.44 | **0.010** | -0.24 | 0.194 | -0.48 | **0.004** | 0.64 | **<0.001** | 0.35 | **0.046** | 0.40 | **0.017** | -0.60 | **<0.001** | 0.32 | 0.069 |
| Plant N uptake | 0.03 | 0.896 | 0.10 | 0.650 | -0.09 | 0.699 | 0.28 | 0.112 | -0.01 | 0.942 | -0.08 | 0.714 | -0.30 | 0.085 | 0.02 | 0.914 |
| Plant C uptake | -0.43 | **0.010** | -0.22 | 0.244 | -0.47 | **0.004** | 0.63 | **<0.001** | 0.35 | **0.046** | 0.41 | **0.015** | -0.59 | **<0.001** | 0.33 | 0.059 |
| pH | -0.32 | 0.069 | -0.35 | **0.046** | -0.27 | 0.133 | 0.41 | **0.017** | 0.22 | 0.244 | 0.22 | 0.242 | -0.33 | 0.059 | 0.19 | 0.353 |
| TOC | 0.09 | 0.713 | 0.13 | 0.542 | -0.04 | 0.882 | 0.00 | 0.987 | 0.07 | 0.749 | 0.04 | 0.882 | -0.03 | 0.896 | 0.08 | 0.714 |
| TN | 0.08 | 0.714 | 0.08 | 0.714 | 0.15 | 0.478 | 0.14 | 0.510 | 0.04 | 0.882 | 0.15 | 0.463 | -0.05 | 0.821 | -0.03 | 0.896 |
| TP | -0.10 | 0.641 | 0.02 | 0.914 | -0.16 | 0.463 | 0.15 | 0.463 | 0.20 | 0.297 | 0.24 | 0.192 | -0.01 | 0.945 | 0.15 | 0.463 |
| TK | -0.12 | 0.568 | -0.09 | 0.705 | -0.12 | 0.568 | 0.02 | 0.942 | 0.19 | 0.353 | 0.08 | 0.713 | -0.13 | 0.542 | 0.19 | 0.353 |
| DOC | -0.36 | **0.042** | -0.11 | 0.593 | -0.24 | 0.196 | 0.42 | **0.014** | 0.35 | **0.046** | 0.44 | **0.010** | -0.29 | 0.101 | 0.47 | **0.004** |
| NH_4_^+^ | 0.31 | 0.082 | 0.29 | 0.094 | 0.21 | 0.266 | -0.14 | 0.510 | -0.13 | 0.542 | -0.12 | 0.584 | 0.26 | 0.140 | -0.15 | 0.463 |
| NO_3_^-^ | 0.44 | **0.010** | 0.27 | 0.133 | 0.33 | 0.059 | -0.37 | **0.040** | -0.35 | **0.046** | -0.32 | 0.069 | 0.31 | 0.082 | -0.13 | 0.522 |
| AP | -0.21 | 0.266 | -0.26 | 0.155 | -0.18 | 0.367 | 0.16 | 0.435 | 0.27 | 0.136 | 0.37 | **0.040** | -0.08 | 0.714 | 0.30 | 0.082 |
| AK | -0.08 | 0.713 | 0.07 | 0.738 | 0.07 | 0.714 | 0.06 | 0.802 | -0.01 | 0.942 | 0.18 | 0.366 | 0.17 | 0.424 | 0.02 | 0.942 |
